# Regulation of *IL-20* Expression by Estradiol through KMT2B-Mediated Epigenetic Modification

**DOI:** 10.1371/journal.pone.0166090

**Published:** 2016-11-02

**Authors:** Chia-Hsin Su, I-Hsuan Lin, Tsai-Yu Tzeng, Wen-Ting Hsieh, Ming-Ta Hsu

**Affiliations:** 1 Institute of Biochemistry and Molecular Biology, School of Life Science, National Yang-Ming University, Taipei 11221, Taiwan, Republic of China; 2 The Center of Translational Medicine, Taipei Medical University, Taipei, Taiwan; 3 VYM Genome Research Center, National Yang-Ming University, University System of Taiwan, Taipei 11221, Taiwan, Republic of China; 4 Chien-Tien Hsu Cancer Research Foundation, Taipei 11221, Taiwan, Republic of China; University of Wisconsin Madison, UNITED STATES

## Abstract

Cytokines are low molecular weight regulatory proteins, or glycoproteins, with both tumor-promoting and inhibitory effects on breast cancer growth. Different cytokines play important roles in breast cancer initiation and progression. Here, we show that of the 39 interleukin (IL) genes, *IL-20* is the only gene over-expressed in MCF-7 cells treated with estradiol (E2) and that induction of *IL-20* expression by estrogen was epigenetically regulated. Methylation of histone H3K4 in the *IL-20* promoter was shown to occur via the specific recruitment of KMT2B by estrogen receptor alpha (ERα), but not by other members of the mixed-lineage leukemia (MLL) family of histone methyltransferases. Depletion of KMT2B, or IL-20, disrupts estrogen signaling, attenuates cell proliferation, reduces colony formation, and results in cell cycle arrest. Furthermore, we demonstrated that KMT2B-mediated epigenetic modification also affected the expression of several ERα target genes. IL-20 and KMT2B expression were also associated with ERα-positive breast cancer tissues. We have revealed an important role for KMT2B in the epigenetic transcriptional regulation of cytokine *IL-20*, and other ERα-responsive genes, in breast cancer cells. Inhibition of IL-20 and KMT2B may have therapeutic benefits in ERα-positive breast cancer.

## Introduction

Breast cancer is the most common malignancy and the principal cause of cancer-related mortality in women. Estrogen receptor (ER), progesterone receptor (PR), and human epithelial growth factor receptor 2 (HER2) are standard clinical tumor markers for determining the appropriate therapy for breast cancer patients [1]. The absence of ER and PR, and the lack of HER2 over-expression have consistently been associated with a poorer prognosis [2]. Inflammatory cytokines are also associated with poor prognosis and reduced survival in patients with breast cancer [3–5]. Cytokines are low molecular weight pleiotropic glycoproteins that play key roles in breast cancer development and progression [6–8]. The role of cytokines in modulating tumor microenvironments has been well studied [9–10], particularly in breast cancer [11,12]. Interleukin (IL)-1, IL-6, IL-11, and transforming growth factor-β (TGF-β) are involved in the stimulation of cancer cell proliferation and invasion [11,13]. Additionally, NF-κB-dependent intracellular signaling and activation of cytokine receptors can accelerate tumor progression [10]. IL molecules play important roles in tumor microenvironments and in activating tumor growth and progression. However, the molecular mechanisms involved in the transcriptional regulation of IL genes in breast cancer have not been widely studied.

Here, we observed that *IL-20* is the sole cytokine over-expressed in ER-positive MCF-7 cells upon estradiol (E2) treatment. *IL-20* was not overexpressed in ER-negative breast cancer cell lines. Analysis of RNA and protein expression also showed overexpression of *IL-20* in ER-positive breast cancer tissues. Induction of *IL-20* expression in E2-treated MCF-7 cells was mediated by epigenetic regulation through the KMT2B histone methyltransferase, but not by other members of the mixed-lineage leukemia (MLL) family of histone methyltransferases.

The MLL gene family is often involved in chromosome translocations in human acute leukemia, causing the fusion of the normal *MLL* gene family member with one of over 60 genes on other chromosomes [14,15,16]. Genes of the *MLL* family (*MLL/KMT2A*, *MLL2/KMT2D*, *MLL3/KMT2C*, *MLL4/KMT2B*, and *MLL5/KMT2E*) encode an evolutionarily conserved family of histone methyltransferases (HMTs). MLL family members regulate the activation of gene expression, including the clustered *HOX* homeobox genes, through methylation of the lysine 4 residue of histone H3 (H3K4) [17–20]. Many *MLL* genes have been described to be involved in different types of cancer, including breast cancers [21–23]. However, the histone methyltransferases responsible for H3K4 methylation of mammalian gene enhancers and promoters remain elusive. The way that HMTs work independently, or cooperatively, with specific transcription factors to epigenetically regulate cell-type-specific gene expression remains to be fully elucidated. Here, we show that KMT2B interacts with ERα to bind the ERα-binding sites of *IL-20* and other ERα target genes with H3K4 modifications. Additionally, depletion of KMT2B or IL-20 led to the inhibition of E2-dependent cell proliferation, loss of colony formation and cell arrest.

## Results

### *IL-20* is induced by estradiol treatment in MCF-7 cells and is strongly associated with ER-positive breast cancer

**Fig 1:**
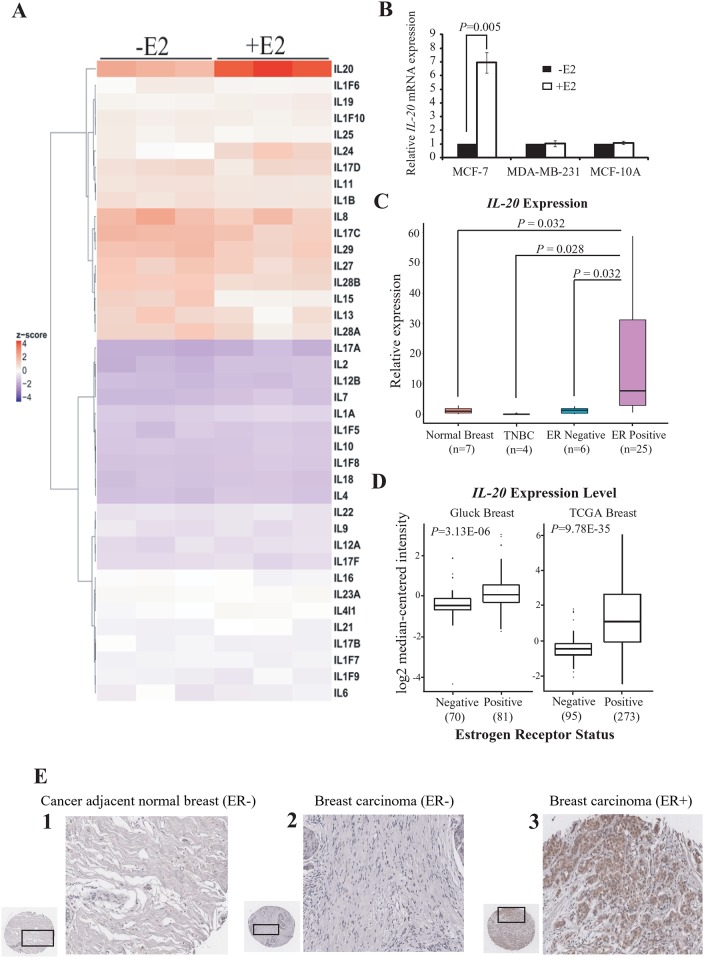
*IL-20* is up-regulated by estradiol treatment in MCF-7 cells and is highly expressed in ER-positive breast cancer. (A) Heat map representation of the gene expression profiles of 39 interleukin (IL) genes in MCF-7 cells with or without E2 treatment for 4 h. (B) Expression of *IL-20* in MCF-7, MAD-MB-231, and MCF-10A cells with or without E2-treatment for 4 h and normalized against 18s rRNA. (C) Quantitative RT-PCR of *IL-20* in seven normal breast tissues, four triple negative breast carcinoma, six ER-negative breast carcinoma, and twenty-five ER-positive breast carcinoma cDNA samples. (Human breast cancer qPCR Array, OriGene). (D) Gluck and TCGA Breast gene expression datasets of *IL-20* mRNA. Data and statistics were obtained from the Oncomine database. (E) IHC analyses of IL-20 protein expression in human breast carcinomas in tissue microarrays (TMAs). The staining results show that ER-positive breast cancer expresses significantly more IL-20 than ER-negative breast cancer, and cancer adjacent normal tissues. Hematoxylin was used as counterstain. Representative images of normal adjacent breast tissue (1), ER-negative malignant breast tissue (2), and ER-positive malignant breast tissue (3) are shown.

We performed genome-wide expression profiling to investigate whether the expression of interleukin (IL) genes was under E2-dependent transcriptional regulation. We used microarray analysis to identify IL gene expression in ER-positive MCF-7 cells with or without E2 induction. Of the 39 IL genes, only *IL-20* was over-expressed in E2-treated MCF-7 cells (Fig 1A). RT-qPCR analysis confirmed that *IL-20* gene expression was significantly induced in E2-treated MCF-7 cells, and was not affected by E2 in the ER-negative cell lines MDA-MB-231 and MCF-10A (Fig 1B). Indeed, ELISA analysis also showed that the amount protein of IL-20 secretion was dramatically induced in E2-stimulated MCF-7 cells (S1 Fig).

We examined *IL-20* mRNA levels across a panel of breast cancer subtypes and normal breast tissues. *IL-20* mRNA levels were significantly elevated in ER-positive breast cancer compared to that in normal breast, triple-negative, and ER-negative breast cancers (Fig 1C). We also showed that *IL-20* was significantly over-expressed in ER-positive breast cancer by using the Gluck and TCGA Breast gene expression datasets available in Oncomine (https://www.oncomine.org/) (Fig 1D).

We further evaluated IL-20 protein levels in an independent tissue microarray panel containing 47 ER-positive breast carcinoma samples, 97 ER-negative breast carcinoma samples, and 6 ER-negative cancer-adjacent normal breast tissue samples. Analysis by immunohistochemistry (IHC) showed that IL-20 was abundantly expressed in 80.9% of ER-positive breast cancer samples (*P* < 0.001). Furthermore, IL-20 expression was observed in only 15.4% of ER-negative breast cancer samples (*P* < 0.001) and in none of the adjacent normal breast tissue samples (Fig 1E). These results indicate that over-expression of *IL-20* mRNA and protein is associated with breast cancer, and particularly with the ER-positive subtype.

### ERα is required for the induction of *IL-20* gene expression

**Fig 2:**
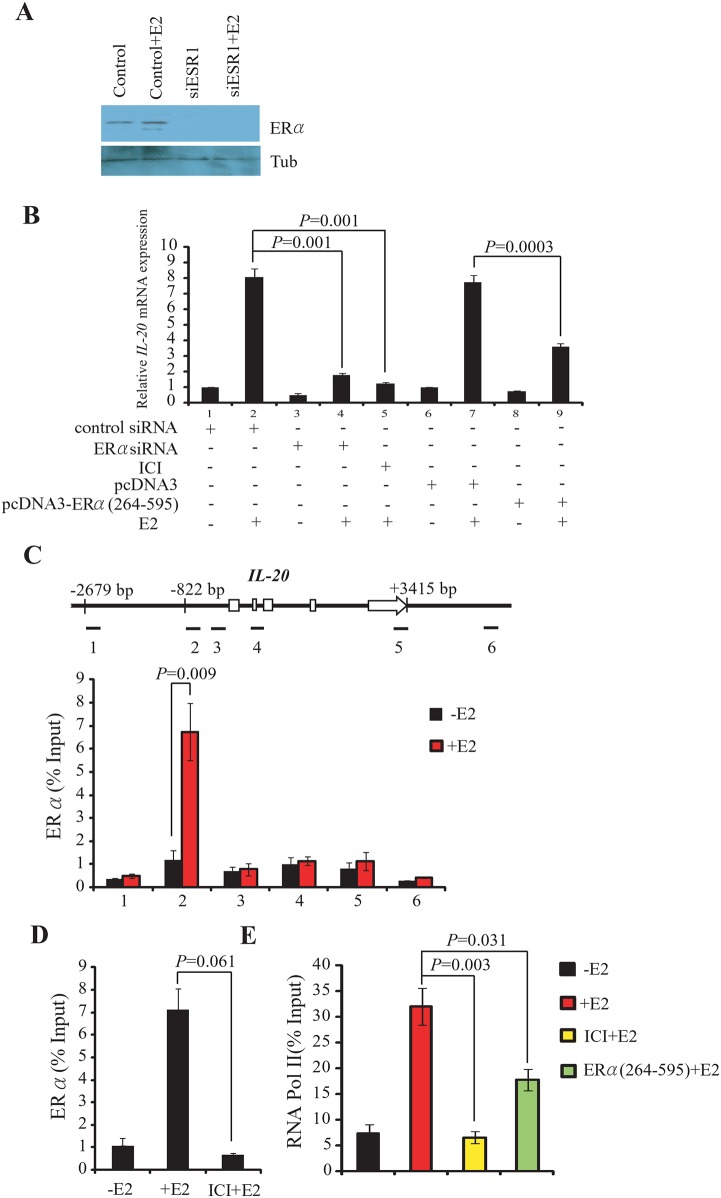
ERα is required for the E2-mediated induction of *IL-20*. (A) Western blot shows the protein level of ERα in cells transfected with the si*ESR1* following E2-stimulation for 30 min. (B) Expression of *IL-20* in MCF-7 cells is dependent on the presence and activity of ERα and E2, and normalized against 18s rRNA. (C) Binding of ERα to the *IL-20* promoter region determined by ChIP assays. Upper panel: Schematic of the *IL-20* locus (exons as open boxes) and the six amplicons (black segments) used in ChIP assays. The specific anti-ERα antibody, HC-20X, was used in the ChIP experiments. Lower panel: Bar chart of the relative levels of ERα at each of the six regions. The mean and SD were calculated from at least two independent experiments. (D) Suppressed ERα binding to the *IL-20* promoter (segment 2) by ICI treatment. The ChIP experiment was carried out in the absence or presence of E2 for 30 min. (E) Inhibition of RNA Pol II binding to the *IL-20* promoter (segment 2) by ICI treatment or over-expression of truncated ERα (ERα_264–595_) determined by ChIP. The experiment was carried out in the presence or absence of E2 for 30 min.

*IL-20* was significantly over-expressed in ER-positive cancer cells and tissues, possibly through estrogen signaling mediated by the ERα estrogen receptor, encoded by *ESR1*. To test this hypothesis, we examined the effect of *ESR1*-specific siRNA, or of the ERα antagonist ICI 182,780 (ICI), on the expression of *IL-20* mRNA MCF-7 cells. *ESR1*-siRNA treatment efficiently depleted ERα with or without E2 treatment (Fig 2A). Depletion of ERα by *ESR1*-siRNA significantly reduced E2-dependent activation of *IL-20* expression (Fig 2B and S2 Fig). Activation of *IL-20* expression was also inhibited by ICI treatment (Fig 2B). Additionally, ELISA analysis confirmed that E2-dependent induction of *IL-20* secretion was significantly decreased in MCF-7 cells transfected with ESR1-siRNAs (S1 Fig).

Transcriptional regulation of *IL-20* by ERα, following E2-dependent activation, was investigated using chromatin immunoprecipitation (ChIP). ChIP assays were performed 0 and 30 min after E2-treatment in MCF-7 cells. We divided the *IL-20* gene body and its flanking sequences into six regions. ChIP experiments were performed to analyze ERα occupancy in these regions with or without E2 treatment. Robust binding of ERα was observed at the promoter region of *IL-20* in E2-treated cells (Fig 2C).

The specific binding of ERα to the *IL-20* promoter following E2 treatment was abolished in the presence of the ERα antagonist, ICI (Fig 2D). Furthermore, over-expression of a truncated ERα, lacking the DNA-binding domain (ERα_264–595_) [24], decreased the induction of *IL-20* expression in E2-induced MCF-7 cells (Fig 2B). As expected, both over-expression of ERα_264–595_ and treatment with ICI inhibited the recruitment of RNA Pol II to *IL-20* chromatin (Fig 2E). These data demonstrate that ERα directly associates with the promoter region of *IL-20* to up-regulate *IL-20* gene expression in the presence of E2.

### Transcriptional induction of *IL-20* by estradiol is dependent on the estrogen response element (ERE)-like sequence in the *IL-20* promoter

We performed motif scanning to examine the ERα binding site of the IL-20 promoter. We revealed a sequence element within segment 2 of the *IL-20* promoter region (5′-**ATGCCA**AAC**AGAGCT**-3′) closely related to the estrogen response element (ERE) (5′-**AGGTCA**CGG**TGACCT**-3′). To examine whether DNA containing this sequence is responsible for E2-mediated transcriptional regulation, we cloned a *Bgl* II—*Nco* I (-1211 to +180 nucleotides) human *IL-20* promoter fragment and transiently transfected it into MCF-7 cells. The ability of the *Bgl* II—*Nco* I *IL-20* promoter fragment to regulate transcription was assessed using a luciferase reporter assay. Compared with controls, treatment with E2 caused a 10.1-fold increase in luciferase activity (S3A Fig). As expected, co-treatment with E2 and ICI abolished E2-induced ERE-driven activity (S3A Fig). In addition, luciferase activity was clearly inhibited when ERα expression was depleted by the *ESR1*-specific siRNA following E2 treatment (S3A Fig).

To further define the sequence element responsible for the E2 response, we transfected MCF-7 cells with clones containing two shorter DNA fragments from the *IL-20* promoter region (-822 to +180 or -499 to +180). Cells transfected with the longer construct (-822 to +180) exhibited a 9.7-fold increase in luciferase activity following E2 treatment, similar to that of the full length construct (S3B Fig). However, cells transfected with the shorter construct (-499 to +180) did not respond to E2 stimulation (S3B Fig). This indicates that the functional ERE-like element is located between -822 and -499 nucleotides upstream from the transcription start site. This is consistent with our ChIP results indicating ERα occupancy at segment 2 of the *IL-20* gene (Fig 2C), and with the location of the ERE-like sequence identified in motif scanning. Furthermore, mutation of the ERE-like element abolished the E2-induced up-regulation of luciferase activity (S3C Fig).

We also performed an *in vitro* oligonucleotide pull-down assay using total protein extract from proliferating MCF-7 cells. We labeled double stranded DNA oligonucleotides corresponding to the perfect ERE, the *IL-20* ERE-like, and mutated *IL-20* ERE-like with biotin. Streptavidin was used to immunoprecipitate proteins associated with each biotin-labeled DNA segment and the immunoprecipitated proteins were examined by western blotting using an anti-ERα antibody. We found that ERα was associated with the perfect ERE and *IL-20* ERE-like sequences, but not with the mutated *IL-20* ERE-like sequence (S3D Fig). These data confirm that ERα binds specifically to the identified ERE-like element in the *IL-20* promoter.

### KMT2B regulates E2-induced *IL-20* over-expression through H3K4 modification

**Fig 3:**
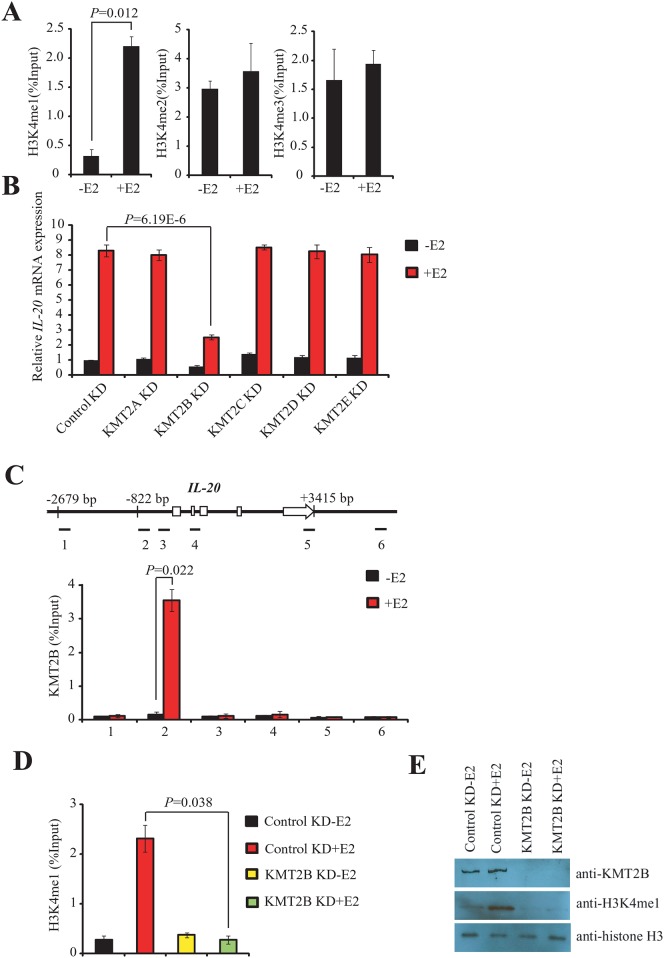
KMT2B regulates E2-mediated induction of *IL-20* gene through the modification of H3K4. (A) Enrichment analysis of the three H3K4 methylations (H3K4me1, H3K4me2, and H3K4me3) at the *IL-20* promoter in MCF-7 cells using ChIP. The experiment was performed with or without E2. The mean and SD were calculated from at least three independent experiments. (B) Expression of *IL-20* as determined by RT-qPCR in KMT2A, KMT2B, KMT2C, KMT2D, or KMT2E-depleted MCF-7 cells in the presence or absence of E2 and normalized against 18s rRNA. (C) Binding of KMT2B to the *IL-20* promoter region as determined by ChIP assays. Upper panel: Schematic of the *IL-20* locus (exons as open boxes) and the six amplicons (black segments). The specific anti-KMT2B antibody, ab104444, was used for the ChIP experiments. DNA isolated from immunoprecipitated chromatin was amplified by qPCR using designed primers. Lower panel: Bar chart showing the relative levels of KMT2B at each of the six *IL-20* gene regions. The mean and SD were calculated from at least two independent experiments. (D) ChIP assays showing the depletion of H3K4me1 at the *IL-20* promoter in KMT2B knockdown cells in the presence or absence of E2. The mean and SD were calculated from at least three independent experiments. (E) Western blotting of nuclear extracts prepared from MCF-7 cells transfected with control siRNA or KMT2B-siRNA in the presence or absence of E2. The antibodies used are shown in the right panel.

We performed ChIP assays using anti-H3K4me1, anti-H3K4me2, and anti-H3K4me3 to determine the nature of histone modifications involved in the epigenetic regulation of *IL-20* expression. Activation mark H3K4me1 was rapidly enriched at the ERα-binding site of *IL-20* 30 min after E2 treatment, whereas there were no significant changes in the levels of H3K4me2 and H3k4me3 (Fig 3A). Furthermore, after 30 min of E2-treatment the level of H3K4me1 declined, and the level of H3K4me3 increased (S4 Fig), suggesting sequential methylation of H3K4.

MLL family members specifically methylate H3K4. Therefore, we examined whether other MLL members were involved in E2-induced *IL-20* gene expression through modification of H3K4 methylation. We used specific siRNAs to separately knockdown the levels of KMT2A (MLL1), KMT2B (MLL4), KMT2C (MLL3), KMT2D (MLL2), and KMT2E (MLL5) following treatment with E2. Depletion of KMT2B significantly suppressed E2-induced expression of *IL-20* (Fig 3B and S2C Fig). siRNA mediated depletion of the other MLL family members investigated (KMT2A, KMT2C, KMT2D, and KMT2E) did not affect E2-induced *IL-20* expression (Fig 3B). The specificity of siRNA-mediated knockdown was confirmed by RT-qPCR (S5 Fig). Indeed, ELISA analysis also showed that E2-dependent induction of *IL-20* secretion was significantly decreased in MCF-7 cells transfected with KMT2B-siRNAs (S1 Fig).

Next, we used ChIP to examine whether KMT2B was recruited to *IL-20* chromatin after E2 treatment. KMT2B was recruited to the ERα binding site of *IL-20* after E2 treatment (Fig 3C). Furthermore, depletion of KMT2B diminished the level of H3K4me1 at the *IL-20* ERα binding site after E2 treatment (Fig 3D). Western blotting and immunostaining showed that depletion of KMT2B significantly reduced global H3K4me1 levels following E2 treatment (Fig 3E and S6 Fig). These findings suggest that KMT2B is recruited to the ERα binding region of the *IL-20* promoter for H3K4 methylation and is required for E2-mediated activation of *IL-20*.

### KMT2B and ERα are interdependently co-localized at the *IL-20* promoter

**Fig 4:**
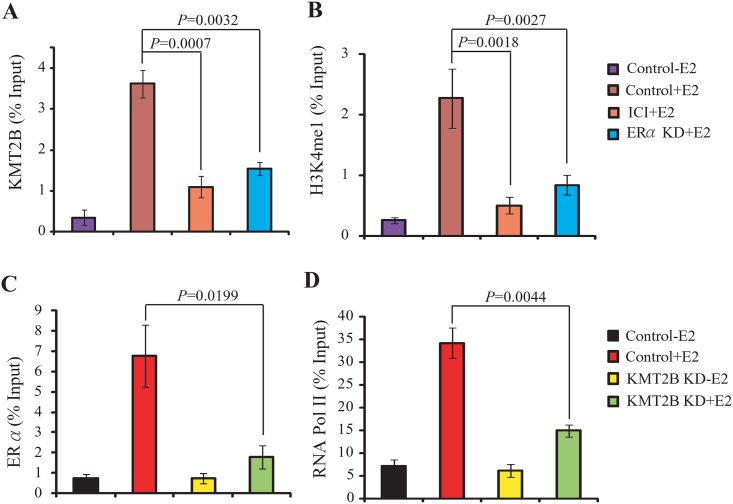
ERα and KMT2B interdependently co-localized at *IL-20* chromatin. (A-B) ChIP analysis of KMT2B recruitment (A) and H3K4me1 enrichment (B) with or without si*ESR1*(ERα knockdown) for 3 days or ICI treatment for 24 h, followed by E2 for 30 min. (C-D) ChIP assay showing the effect of KMT2B depletion on the recruitment of ERα (C) and RNA Pol II (D). The primers that amplify the ERα binding region of *IL-20*, i.e. segment2, were used in these experiments.

Since KMT2B was recruited to the ERα binding site of *IL-20* promoter, we examined whether the recruitment of KMT2B to the ERα binding site was ERα-dependent. Depletion of ERα, or ICI treatment, abolished KMT2B recruitment to *IL-20* chromatin in E2-treated cells (Fig 4A). Additionally, depletion of ERα, or ICI treatment, decreased the level of H3K4me1 in E2-treated cells (Fig 4B). Conversely, in E2-treated cells depletion of KMT2B abolished recruitment of ERα and RNA polymerase II (RNA Pol II) to *IL-20* chromatin (Fig 4C and 4D). These data suggest that ERα and KMT2B are interdependently associated with *IL-20* chromatin.

**Fig 5:**
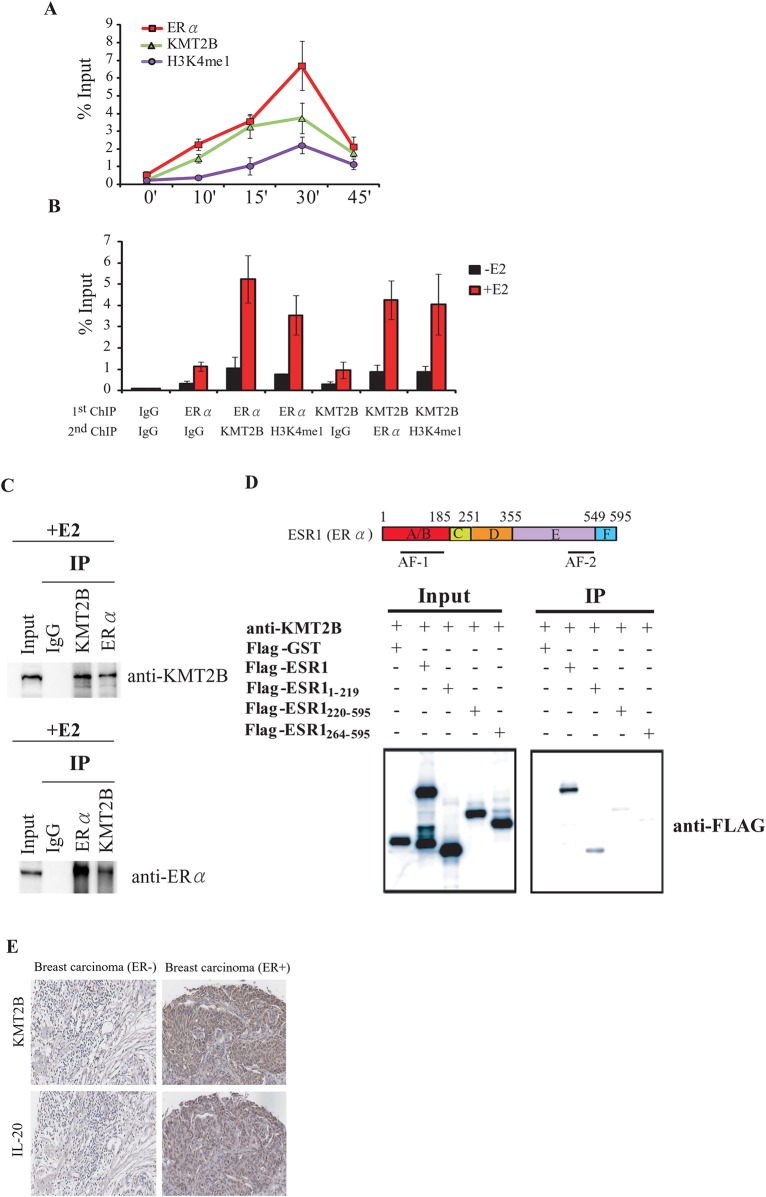
KMT2B and ERα form a complex at the promoter of the *IL-20* gene. (A) Kinetic ChIP experiments were performed using KMT2B, H3K4me1, and ERα specific antibodies. A single chromatin was prepared for ChIP assay at each time point. (B) ChIP-ReChIP to determine the KMT2B and ERα co-occupancy at the *IL-20* promoter. Chromatin was prepared from MCF-7 cells treated with E2 for 30 minutes and then subjected to the ChIP procedure using the antibodies labeled as "1^st^ ChIP.” The second immunoprecipitation was carried out using the antibodies labeled as "2^nd^ ChIP.” (C) Co-immunoprecipitation of endogenous KMT2B and ERα. MCF-7 cells were treated with E2 for 24 h, and whole-cell lysates were immunoprecipitated using KMT2B or ERα antibodies. Western blotting was performed on the immunoprecipitated proteins using anti-KMT2B or anti-ERα. (D) Upper panel: Schematic of the ERα functional domains. Lower panel: Immunoprecipitation analysis of the ERα functional domains that interact with KMT2B. Interactions between the endogenous KMT2B and the *in vivo* transcribed/translated Flag-tagged ERα fragment were confirmed by an immunoprecipitation assay using anti-KMT2B antibody followed by western blotting with anti-FLAG antibody. (E) KMT2B and IL-20 protein expression in ER-positive and ER-negative breast cancer assayed by IHC.

We performed ChIP analysis at 0, 10, 15, 30, and 45 min after E2 induction to establish the order in which KMT2B and ERα are recruited to the promoter region of *IL-20*. KMT2B and ERα were rapidly recruited to the *IL-20* promoter region and kinetic ChIP analysis showed coordinated binding of KMT2B and ERα to the promoter after E2 induction (Fig 5A).

To determine whether KMT2B and ERα are recruited to the *IL-20* promoter as a complex we performed ChIP-ReChIP experiments after 30 min of E2 treatment. KMT2B and ERα were found to be present together at the *IL-20* promoter in an E2-dependent manner (Fig 5B). Additionally, H3K4me1 was found co-localized with either KMT2B or ERα at the *IL-20* promoter in an E2-dependent manner (Fig 5B). These findings suggest that the KMT2B and ERα form a protein complex at the *IL-20* promoter to regulate *IL-20* expression. Physical interaction between KMT2B and ERα was verified by co-immunoprecipitation in E2-induced MCF-7 cells (Fig 5C). Furthermore, using a FLAG-tagged *in vitro* pull-down assay, we showed that KMT2B directly interacts with an ERα fragment containing the activation function 1 (AF1) domain (Fig 5D).

Following E2 treatment, KMT2B interacts with ERα to regulate *IL-20* expression. Therefore, we assessed KMT2B expression according to the ER status of clinical tissue samples. To confirm the role of KMT2B in breast cancer, we performed IHC on tissue microarrays to observe the *in situ* expression of the KMT2B protein. KMT2B expression was significantly associated with ERα-positive breast cancer (70.2%; *P* < 0.001, Fig 5E). Of the 53 KMT2B-positive samples, 33 were ERα-positive. Furthermore, of the 33 KMT2B-positive / ERα-positive samples, 29 were positive for IL-20 (87%; *P* < 0.001, Fig 5E). These data suggest that KMT2B and ERα synergistically regulate IL-20 in breast cancer.

### ERα and KMT2B-mediated induction of *IL-20* expression is required for E2-stimulated cell proliferation

**Fig 6:**
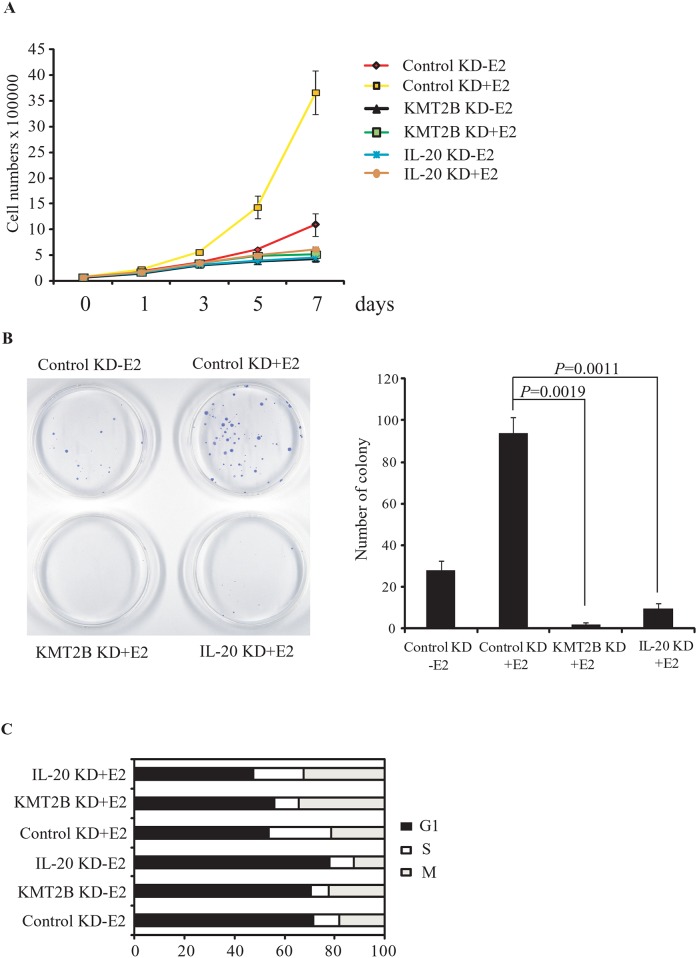
The effect of KMT2B and IL-20 knockdown on cell proliferation in E2-induced MCF-7 cells. (**A**) Cell proliferation assays and (**B**) colony formation assays of KMT2B or IL-20 depleted MCF-7 cells. Cells were transfected with control siRNA, siKMT2B, or siIL-20 in the absence or presence of E2. (C) Cell-cycle analysis of KMT2B or IL-20 depleted MCF-7 cells using propidium iodide staining and flow cytometry. MCF-7 cells were transfected with control siRNA, si*KMT2B*, and si*IL-20* in the absence or presence of E2.

Here, we have shown that the E2-mediated induction of *IL-20* expression is regulated by KMT2B and ERα. E2 also binds to ERα to induce cell proliferation. Therefore, we examined whether KMT2B or IL-20 were involved in the E2-dependent proliferation of MCF-7 cells. KMT2B or IL-20 knockdown inhibited the proliferation of MCF-7 cells (Fig 6A). Furthermore, in colony formation assays, depletion of either KMT2B or IL-20 significantly inhibited colony formation, as seen by the reduced number and size of MCF-7 colonies (Fig 6B). We further examined the effect of KMT2B or IL-20 knockdown on E2-induced cell proliferation using flow cytometry. Depletion of KMT2B or IL-20 in E2-treated MCF-7 cells resulted in M-phase cell cycle arrest (Fig 6C). These data suggest that the induction of *IL-20* by KMT2B is required for E2-induced proliferation in ERα-positive cells.

### KMT2B plays a critical role in regulating E2-dependent transcription in MCF-7 cells

To elucidate the effect of KMT2B knockdown on genes expression in E2-induced MCF-7 cells we performed transcriptome analysis using microarrays. Of the 369 genes up-regulated by E2 treatment, 195 (52.8%) were down-regulated upon KMT2B depletion (S7A Fig). We used RT-qPCR to confirm the microarray results for five ERα target genes: *PGR*, *BCL2*, *GREB1*, *SIAH2*, and *TFF1*. E2-dependent induction of *PGR*, *BCL2*, *GREB1*, *SIAH2I*, and *TFF1* was significantly suppressed in KMT2B-depleted cells (S7B Fig).

We next analyzed the mechanism by which KMT2B regulates ERα target genes. First, we performed ChIP assays using an anti-ERα antibody to confirm E2-dependent ERα recruitment to the promoters of *BCL2*, *GREB1*, and *TFF1* in MCF-7 cells. In E2-induced cells, ERα was bound to the ERα binding sites of the *BCL2* enhancer, and the *GREB1* and *TFF1* promoters (S7C Fig). Additionally, ChIP using an anti-KMT2B antibody revealed that KMT2B was bound to the same regulatory regions of these genes (S7D Fig). E2-dependent ERα binding was abolished when KMT2B was depleted, and *vice versa*. In the presence of E2, the ERα binding sites were highly enriched with H3K4me1, but became depleted when ERα or KMT2B were knocked-down (S7E Fig). KMT2B and ERα are both required for the E2-induced deposition of active H3K4me1 mark and activation of ERα target genes. Therefore, the *cis*-regulatory elements of *BCL2*, *GREB1*, and *TFF* are able to recruit both KMT2B and ERα. Finally, KMT2B and ERα are also required for the recruitment of RNA Pol II to gene promoters in E2-treated MCF-7 cells (S7F Fig). These data demonstrate that ERα co-operates with KMT2B to epigenetically and transcriptionally regulate the E2-mediated induction of ERα target genes.

## Discussion

Breast cancer is the most common type of cancer in woman. The initiation and progression of breast cancer has been under intensive investigation. Inflammation has emerged as a mediator of pro-tumorigenic processes and tumorigenesis. The secretion of cytokines, chemokines, and growth factors by infiltrating immune cells and activated fibroblasts results in an inflammatory environment that can contribute to cancer development [25–27]. Recently, several cytokines have been identified as regulators involved in the inflammatory tumor microenvironment [10, 28–30]. Here, we showed that cytokine IL-20 is an ERα target associated with ER-positive breast cancer. IL-20 family cytokines facilitate communication between epithelial and leukocytes [31] and IL-20 is involved in psoriasis, stroke and rheumatoid arthritis.

Recently IL-20 has been shown to have roles in breast and colon cancers [8,30]. Hsu *et al*. found that *IL-20* expression was associated with advanced breast cancer and that an IL-20 monoclonal antibody could suppress tumor progression and bone metastasis in a mouse model [30]. Here, we found that *IL-20* is more specifically associated with ER-positive breast cancer and is regulated by estrogen. Given that Hsu *et al*. did not analyze the ER status of their breast cancer tissue samples, it is not clear how many of the IL-20 positively tumors were ER-positive. Our IHC analyses showed that the majority of IL-20 positive breast cancer tissues we examined are also ER-positive. However, we did identify some ER-negative breast cancer tissues with IL-20 expression, and the nature of these tumors remains to be characterized.

Recently, Lin *et al*. applied the chromatin immunoprecipitation paired-end diTags (ChIP-PET) technique in MCF-7 cells to identify the ERα binding sites of the whole genome [32]. Of the 1,234 high quality ERα binding sites identified, 71% were ERE-like sites, 25% were putative half-ERE sites, and 4% had no recognizable resemblance to ERE sequences. By integrating gene expression profiling with high-throughput ChIP assays, several reports have indicated that ERα binding to ERE-like sequences is responsible for gene activation [32,33]. Here, a novel imperfect palindromic ERE was found in the *IL-20* gene promoter. This promoter was activated by E2-stimulation through the E2-dependent binding of ERα to this imperfect palindromic ERE. Treatment with the ER antagonist ICI abolished both the E2-dependent ERα binding to the ERE-like element and recruitment of RNA Pol II. These results confirm the specificity of the ERα binding to the ERE-like element identified in this study. Furthermore, over-expression of truncated ERα, lacking the DNA binding domain, abolished E2-stimulated induction of *IL-20*.

Luciferase activity was abrogated in the mutated ERE-like element of *IL-20* after E2-treatment. Furthermore, the ERE-mutant did not bring down ERα protein in an *in vitro* oligonucleotide pull-down assay. Results from the earlier genome-wide ChIP-based sequencing analysis performed in MCF-7 cells have indicated that the *IL-20* promoter contains a putative ER*α* binding site [34,35], and this coincides with the ERE-like element identified in this study. Herein, we demonstrated that estradiol (E2)-stimulation of *IL-20* over-expression occurs through an ERα-mediated mechanism. E2-liganded ERα binds directly to the *IL-20* ERE-like element and recruits co-activator proteins and the RNA Pol II transcription machinery to enhance transcription.

We showed that the epigenetic regulation of *IL-20* transcription is mediated by the binding of KMT2B to the AF1 domain of ERα. KMT2B is a member of the MLL family, known to be involved in infantile leukemia and tumor cell proliferation [36]. Significantly, no other MLL members were recruited for the E2-dependent activation of *IL-20* expression. Transcriptome analysis revealed that the expression of over half of the ERα target genes was affected by KMT2B depletion following E2 treatment. This indicates that KMT2B plays a specific and critical role in the regulation of ERα signaling. Indeed, analysis of three ERα target genes showed that the *cis*-regulatory elements were bound by KMT2B and regulated by KMT2B-mediated H3K4 modification.

ERα transcriptional activation requires the recurrent binding of multiple co-regulators onto its target gene promoters in the presence of continuous estrogen stimulation. The dynamic assembly and dissolution of co-regulators from estrogen responsive promoters following E2-liganded ERα activation and deactivation turns the basal transcription machinery ‘on’ and ‘off’, respectively [37,38]. Within 30 min of the addition of estrogen, co-regulators such as SRC-1, AIB1, H2A.Z, H2ac, and p300 are rapidly recruited to ERα binding sites to activate the expression of ERα target genes [24,37,38]. Furthermore, ERα can recruit histone demethylase enzymes KDM3A, KDM4B, and KDM6B to ERα targeted chromatin to assert histone modification changes [39–41]. Herein, we demonstrated that a histone methyltranferase, KMT2B, plays a significant role in enhancing the transcriptional activity of ERα through the enrichment of active H3K4 methylation. ChIP analysis showed that knockdown of KMT2B reduced H3K4 methylation, and abrogated the recruitment of ERα to *cis*-regulatory elements adjacent the promoter regions of the ER-target genes *IL-20*, *BCL2*, *TFF1*, and *GREB1*. Further investigation using whole-genome approaches is necessary to fully elucidate the roles of KMT2B and IL-20 in breast cancer carcinogenesis.

We describe that KMT2B and ERα, along with H3K4me enrichment at the promoter, regulate the transcription of ERα target genes, including *IL-20*, upon E2 treatment. Depletion of *IL-20* or KMT2B in E2-stimulated MCF-7 cells abrogated the over-expression of *IL-20* mRNA and halted cell proliferation. This suggests that these molecules have potential application in breast cancer diagnosis and as targets for cancer intervention.

## Materials and Methods

### Cell culture and transfection

MCF-7 cells were maintained in RPMI 1640 medium supplemented with 10% fetal bovine serum (FBS). For treatment of cells with 17β-estradiol (E2), MCF-7 cells were grown in RPMI 1640 medium without Phenol Red (Gibco) and supplemented with 5% charcoal-dextran-treated FBS for at least 3 days. E2 and ICI 182,780 (Sigma) were used at concentrations of 10 nM and 100 nM, respectively. MCF-10A cells were maintained in DMEM/F12 (1:1) medium supplemented with 20 ng/ml epidermal growth factor, 100 ng/ml cholera toxin, 0.01 mg/ml insulin, 500 ng/ml hydrocortisone, and 5% horse serum (Sigma). MDA-MB-231 cells were maintained in DMEM medium supplemented with 10% FBS. Cells were transfected using Lipofectamine^®^ RNAiMAX (Invitrogen) according to the manufacturer's instructions.

### siRNA knockdown and quantitative PCR analysis

siRNA targeting *ESR1* (siESR1:SASI_Hs01_00078593; siESR1’:SASI_Hs01_00078594; siESR1”:SASI_Hs01_00078595), *KMT2A* (SASI_Hs01_00090459), *KMT2B* (siKMT2B:SASI_Hs01_00240396; siKMT2B’:SASI_Hs01_00240397; siKMT2B”:SASI_Hs01_00240398), *KMT2C* (SASI_Hs01_00037084), *KMT2DE* (SASI_Hs01_00145443) and *KMT2E* (SASI_Hs01_00238555) were obtained from MISSION RNA (Sigma-Aldrich, St. Louis, MO). Cells were transfected with siRNA and Lipofectamine^®^ RNAiMAX (Invitrogen) for 72 h according to the manufacturer's instructions. MISSION^®^ siRNA Universal Negative Control (Sigma-Aldrich) was used as knockdown control.

Total RNA was isolated from cells transfected with siRNA as indicated. Quantitative PCR (qPCR) was performed using SYBR Green dye on a Roche Applied Science LightCycler^®^ 2.0 Real-Time PCR System. Briefly, total RNA was reverse transcribed into cDNA using SuperScript III (Invitrogen, Carlsbad, CA) in the presence of random hexamers (Invitrogen). All reactions were performed in triplicates with SYBR green master mix (Sigma) and 20M of both the forward and reverse primers according to the manufacturer's recommended thermocycling conditions. Then the PCR products were subjected to melting curve analysis. Finally, the relative gene expression ratio of target genes to 18S rRNA for each sample was calculated. The primer sequences are listed in S1 Table.

### RNA extraction and Affymetrix microarray analysis

Total RNA was extracted using Trizol reagent (Sigma-Aldrich; St. Louis, MO). A Human U133 plus 2.0 (Affymetrix) was used for Affymetrix microarray analysis.

### Transcriptome analysis

The microarray raw data deposited in ArrayExpress (accession E-MTAB-4923) was preprocessed with the justRMA function of the affy Bioconductor package [42] with GeneAnnot based custom CDF (gahgu133plus2 v2.2.1) [43]. The log-2 normalized intensities were adjusted for batch effects by using ComBat function of the sva Bioconductor package [44]. The cmdscale function was used for multidimensional scaling (MDS) analysis before and after batch correction. The analyses were performed within the R environment. Two independent breast cancer datasets, TCGA Breast and Gluck Breast, hosted by the Oncomine database (http://www.oncomine.org) were used for differential expression analysis of *IL20* of ER-positive and ER-negative tumors in a clinical setting [45].

### Transcription factor binding sites (TFBS) analysis

The promoter nucleotide sequence of *IL20* was obtained from UCSC Genome Browser. In the R environment, the promoter sequence was scanned for potential ESR1 motifs using the searchSeq function of the TFBSTools Bioconductor package [46]. The position weight matrices of ESR1, namely MA0112.1, MA0112.2 and MA0112.3, were obtained from the JASPAR2016 Bioconductor data package. Predicted sites that had negative scores or relative scores of less than 0.7 were subsequently removed.

### Immunohistochemistry (IHC)

IHC staining of IL-20 and KMT2B proteins were performed on a breast cancer tissue array (BR1503c, US Biomax). The slides containing tissue sections were baked at 60°C for at least 30 min to melt the paraffin, followed by deparaffinization in xylene and rehydration in graded alcohol into water. The slides were soaked in antigen retrieval buffer (pH 9.0, Dako) and heated in microwave oven for 10 min twice under defrosting condition. The slides were washed with 1X PBS and 3% hydrogen peroxide for 5 min to inactivated endogenous peroxidase activity. The slides were further incubated with blocking reagent (5% BSA in 1X PBS) for 1 h at room temperature. Subsequently, the slides were washed three times with 1X PBS and incubated with primary antibodies against IL-20 (sc-134365) (Santa Cruz Biotechnology, Inc) and KMT2B (ab104444) (abcam) at 4°C for 16 to 18 h. The slides were washed three times with 1X PBS to remove unbound antibodies and incubated with Dako REAL EnVision^™^/HRP, Rabbit/Mouse (ENV) (Dako, Glostrup, Denmark) for 20 min at room temperature. Bound primary antibodies were detected with the Dako REAL^™^ EnVision^™^ Detection System. Stained sections were counterstained with Mayer's hematoxylin, dehydrated and coverslips were mounted using mounting solution for microscopic analysis.

### Immunoprecipitation

Cells were transiently transfected with expression plasmids by using LipofectAMINE reagent (Invitrogen). Cells were harvested 36–48 h after DNA transfections for co-immunoprecipitation experiments. Cells were lysed in lysis buffer. Cleared cellular extracts were then incubated with anti-FLAG at 4°C for 2–4 h. The immunoprecipitated proteins were resolved by 8% SDS-PAGE, transferred onto a PVDF membrane. Western blot was performed using antibodies as indicated.

### *In vitro* pull down

FLAG fusion proteins were created using an EasyXpress Protein Synthesis kit (QIAGEN) according to the protocol of the manufacturer. Briefly, *in vitro* translated proteins were incubated with anti-FLAG/beads complex at 4°C overnight. The beads were then washed five times in 500 μl of 0.5% NP-40/PBS washing buffer, followed by boiled in 20 ml 2X sample buffer. After this the samples were analyzed by immunoblotting.

### Chromatin Immunoprecipitation (ChIPs) and Re-ChIPs Assays

The ChIP and Re-ChIP assays were performed as described previously [24]. Briefly, Cell lysates were prepared, sonicated, and immunoprecipitated with anti-KMT2B (ab104444; abcam), anti-ERα (HC-20X; Santa Cruz Biotechnology, Inc), anti-RNA Pol II (05-623B; Millipore) or anti-H3K4me1 (07–436; Millipore) antibodies. The protein-DNA cross-linking of the immunoprecipitated complexes were reversed and the DNA was extracted for subsequent Real-Time qPCR analysis. The primer sets for the qPCR are listed in S2 Table. For the Re-ChIP experiments, after the initial ChIP with the first antibody, the protein-DNA complexes were eluted by incubation for 30 min at 37°C in 25 μl 10 mM DTT. After centrifugation, the supernatant was diluted 20 times with Re-ChIP buffer and subjected again to the ChIP procedure with the second antibody.

### ELISA

Interleukin-20 concentrations were determined in culture supernatants by enzyme-linked immunosorbant assay (ELISA) kit, according to the instructions provided by manufacturer (Quantikine human IL-20, R&D Systems, Minneapolis, MN, USA). The IL-20-EASIA is a solid phase Enzyme Amplified Sensitivity Immunoassay performed on microtiterplate. The plates were read at O.D. 450 nm in a microplate reader.

### Luciferase Reporter Assays

The three serial deletion of 5′ UTR of *IL-20* were amplified by PCR from genomic DNA extracted from MCF7 cells with *Bgl* II and *Nco* I linkers. These fragments were directionally cloned upstream of the Renilla luciferase ORF of the pGL3-basic vector that also contains a constitutively expressed firefly luciferase gene, which is used to normalize transfections. All constructs were confirmed by sequencing. MCF-7 cells were plated into 12- well plates and cotransfected with 0.5 μg pCMV-β-galactosidase reporter vectors in absence or presence of E2 using Lipofectamine 2000 (Invitrogen). Luciferase activity was measured using the Dual-Glo Luciferase Assay System (Promega). Experiments were performed in triplicate wells of a 12- well plate and were repeated at least three times.

### *In vitro* oligonucleotide pull-down assay

*In vitro* oligonucleotide pull-down assay was performed as described previously [47]. These 5′ biotin modified double-stranded oligonucleotides sequence containing a perfect ERE (5’-biotin-CCTTGGTGTCGCGGGTCATAATGACC GGAGCTTTTCCC-3’) [47], an ERE-like of IL-20 (5’-biotin-ATCTCAGACAAA TGCCAAACAGAGCTCAGTTTCTCTGC-3’) and the ERE-like mutant of IL-20 (5’-biotin-ATCTCAGACAAACTCCAAACAGAGTTCAGTTTCTCTGC-3’) complementary sense and antisense oligos were annealed for *in vitro* oligonucleotide pull-down assay.

### Statistical analysis

All statistical analyses were performed using the two-tailed Students *t*-tests. Error bars represent standard deviations.

## Supporting Information

S1 Fig IL-20 expression levels determined by ELISA. ELISA measurements of IL-20 levels in the culture medium from MCF-7 cells transfected with *ERα* or *KMT2B* siRNAs following E2-stimulation.
(DOCX)

S2 Fig *IL-20* expression by RT-qPCR assay in MCF-7 cells treated with other *ESR1* or *KMT2B* siRNAs. (A) RT-PCR asay shows the mRNA level of ERα in cells transfected with the *ERα siRNAs* following E2-stimulation for 4 hours. (B) RT-PCR asay shows the mRNA level of KMT2B in cells transfected with the *KMT2B siRNAs* following E2-stimulation for 4 hours. (C) Expression of *IL-20* in MCF-7 cells is dependent on the presence and activity of ERα, KMT2B and E2, and normalized against 18s rRNA.
(DOCX)

S3 Fig Luciferase activity analysis of plasmid clones containing ERE-like element of *IL-20* promoter. (A) MCF-7 cells were transiently transfected with luciferase fusion vectors containing 1391 bp of the flanking DNA relative to the *IL-20* transcriptional start site. As indicated, transfected cells were treated with 10 nM E2, 1μM ICI or si*ESR1* for 24 hours and luciferase activity was determined. (B) Mapping of sequence element responding to E2 treatment. Specific fragments of the promoter region of the *IL-20* were cloned upstream of luciferase cDNA in the pGL3-basic vector, and were transiently transfected into MCF-7 cells in the presence or absence of E2. (C) Mutation analysis of the ERE-like. The luciferase fusion vector containing the control and mutated ERE-like sequence of the *IL-20* promoter region was transfected into MCF-7 cells in the absence or presence of E2. (D) An *in vitro* oligonucleotide pull-down assay to demonstrate the binding of ERα to the ERE-like sequence of *IL-20* promoter region. The assay was performed using biotinylated 38-bp double-stranded oligonucleotides containing a perfect ERE, an ERE-like of *IL-20* and the ERE-like mutant.
(DOCX)

S4 Fig Kinetic ChIP experiments were performed using H3K4me1, H3K4me2 and H3K4me3 specific antibodies. Cells were treated with 2.5 mM α-amanitin for 2 h followed with 10 nM E2 treatment to carry out the kinetic ChIP assay. A single chromatin was prepared for ChIP assay at each time point.
(DOCX)

S5 Fig Expression of KMTs was determined by RT-qPCR in KMT2A, KMT2B, KMT2C, KMT2D and KMT2E-depleted MCF-7 cells and normalized against 18s rRNA. (DOCX)

S6 Fig H3K4me1 immunostaining (red) in Control knockdown or KMT2B knockdown MCF-7 cells in the presence of E2. (DOCX)

S7 Fig KMT2B regulates E2-dependent genes transcription in MCF-7 cells. (A) A Venn diagram showing E2-stimulated genes down-regulated by KMT2B knockdown in MCF-7 cells. (B) Expression levels of *PGR*, *BCL2*, *GREB1*, *SIAH2*, and *TFF1* in KMT2B-depleted MCF-7 cells with or without E2 for 4 h. Expression levels were normalized against 18S rRNA. (C) ChIP assay showing the effect of KMT2B depletion on the E2-dependent recruitment of ERα at *BCL2*, *GREB1*, and *TFF1* chromatin. (D) ChIP assays showing the effect of ERα depletion on the E2-dependent recruitment of KMT2B at *BCL2*, *GREB1*, and *TFF1* chromatin. ChIP assays showing the effect of ERα or KMT2B depletion on the enrichment of H3K4me1 mark (E) and the recruitment of RNA Pol II (F) at *BCL2*, *GREB1*, and *TFF1* chromatin.
(DOCX)

S1 Table Primers for qRT-PCR assay. (DOCX)

S2 Table Primers for chromatin immunoprecipitation assay. (DOCX)
