# Supplementary material for: Regulation of IL-20 Expression by Estradiol through KMT2B-Mediated Epigenetic Modification
Source: PLoS One. 2016 Nov 2;11(11):e0166090. doi: 10.1371/journal.pone.0166090 (PMC5091760; doi:10.1371/journal.pone.0166090)
Supplement: S1 Fig — ELISA measurements of IL-20 levels in the culture medium from MCF-7 cells transfected with ERα or KMT2B siRNAs following E2-stimulation. (DOCX) [file pone.0166090.s001.docx]

**S1 Fig**


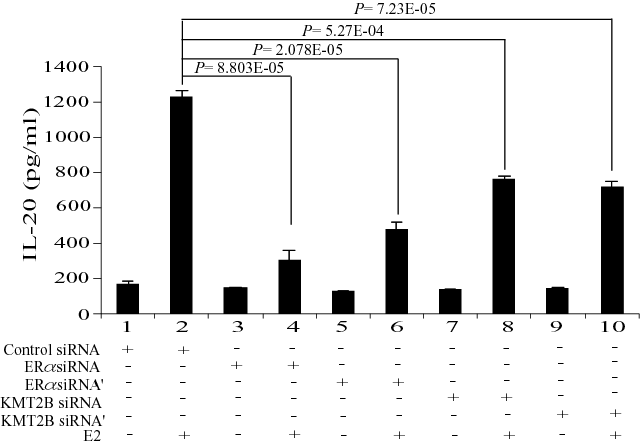


**S1 Fig. IL-20 expression levels determined by ELISA.** ELISA measurements of IL-20 levels in the culture medium from MCF-7 cells transfected with *ERα* or *KMT2B* siRNAs following E2-stimulation.
