# Supplementary material for: Regulation of IL-20 Expression by Estradiol through KMT2B-Mediated Epigenetic Modification
Source: PLoS One. 2016 Nov 2;11(11):e0166090. doi: 10.1371/journal.pone.0166090 (PMC5091760; doi:10.1371/journal.pone.0166090)
Supplement: S2 Fig — (A) RT-PCR asay shows the mRNA level of ERα in cells transfected with the ERα siRNAs following E2-stimulation for 4 hours. (B) RT-PCR asay shows the mRNA level of KMT2B in cells transfected with the KMT2B siRNAs following E2-stimulation for 4 hours. (C) Expression of IL-20 in MCF-7 cells is dependent on the presence and activity of ERα, KMT2B and E2, and normalized against 18s rRNA. (DOCX) [file pone.0166090.s002.docx]

**S2 Fig**


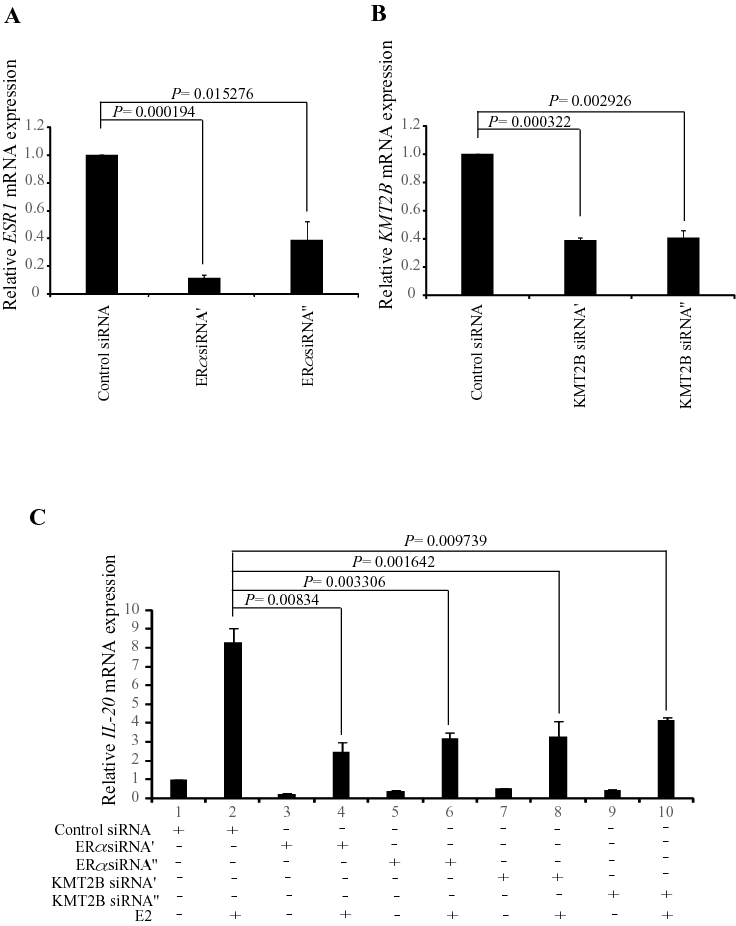


**S2 Fig**. ***IL-20* expression by RT-qPCR assay in MCF-7 cells treated with other ESR1 or KMT2B siRNAs.** (A) RT-PCR asay shows the mRNA level of ERα in cells transfected with the *ERα siRNAs* following E2-stimulation for 4 hours. (B) RT-PCR asay shows the mRNA level of KMT2B in cells transfected with the *KMT2B siRNAs* following E2-stimulation for 4 hours.(C) Expression of *IL-20* in MCF-7 cells is dependent on the presence and activity of ERα, KMT2B and E2, and normalized against 18s rRNA.
