# Supplementary material for: Regulation of IL-20 Expression by Estradiol through KMT2B-Mediated Epigenetic Modification
Source: PLoS One. 2016 Nov 2;11(11):e0166090. doi: 10.1371/journal.pone.0166090 (PMC5091760; doi:10.1371/journal.pone.0166090)
Supplement: S3 Fig — (A) MCF-7 cells were transiently transfected with luciferase fusion vectors containing 1391 bp of the flanking DNA relative to the IL-20 transcriptional start site. As indicated, transfected cells were treated with 10 nM E2, 1μM ICI or siESR1 for 24 hours and luciferase activity was determined. (B) Mapping of sequence element responding to E2 treatment. Specific fragments of the promoter region of the IL-20 were cloned upstream of luciferase cDNA in the pGL3-basic vector, and were transiently transfected into MCF-7 cells in the presence or absence of E2. (C) Mutation analysis of the ERE-like. The luciferase fusion vector containing the control and mutated ERE-like sequence of the IL-20 promoter region was transfected into MCF-7 cells in the absence or presence of E2. (D) An in vitro oligonucleotide pull-down assay to demonstrate the binding of ERα to the ERE-like sequence of IL-20 promoter region. The assay was performed using biotinylated 38-bp double-stranded oligonucleotides containing a perfect ERE, an ERE-like of IL-20 and the ERE-like mutant. (DOCX) [file pone.0166090.s003.docx]

**S3 Fig**


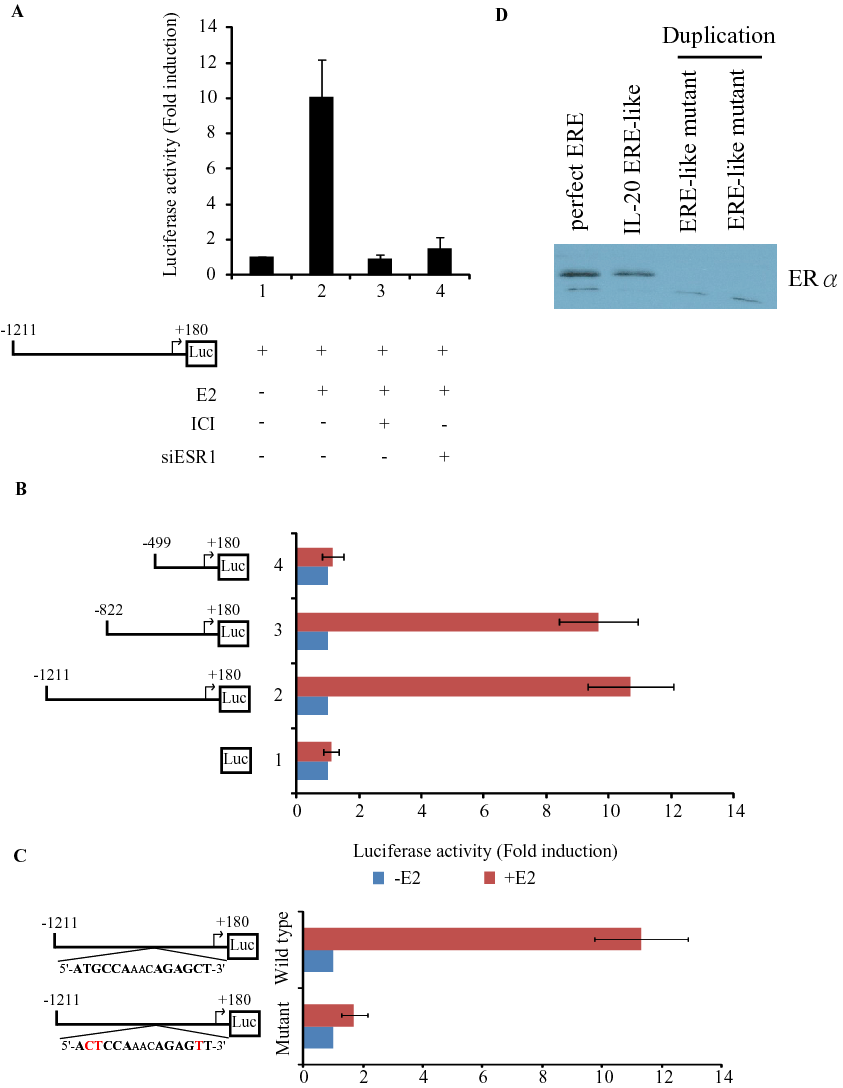


**S3 Fig. Luciferase activity analysis of plasmid clones containing ERE-like element of *IL-20* promoter.** (A) MCF-7 cells were transiently transfected with luciferase fusion vectors containing 1391 bp of the flanking DNA relative to the *IL-20* transcriptional start site. As indicated, transfected cells were treated with 10 nM E2, 1μM ICI or siESR1 for 24 hours and luciferase activity was determined. (B) Mapping of sequence element responding to E2 treatment. Specific fragments of the promoter region of the *IL-20* were cloned upstream of luciferase cDNA in the pGL2-basic vector, and were transiently transfected into MCF-7 cells in the presence or absence of E2. (C) Mutation analysis of the ERE-like. The luciferase fusion vector containing the control and mutated ERE-like sequence of the *IL-20* promoter region was transfected into MCF-7 cells in the absence or presence of E2. (D) An *in vitro* oligonucleotide pull-down assay to demonstrate the binding of ERα to the ERE-like sequence of *IL-20* promoter region. The assay was performed using biotinylated 38-bp double-stranded oligonucleotides containing a perfect ERE, an ERE-like of *IL-20* and the ERE-like mutant.
