# Supplementary material for: Regulation of IL-20 Expression by Estradiol through KMT2B-Mediated Epigenetic Modification
Source: PLoS One. 2016 Nov 2;11(11):e0166090. doi: 10.1371/journal.pone.0166090 (PMC5091760; doi:10.1371/journal.pone.0166090)
Supplement: S4 Fig — Cells were treated with 2.5 mM α-amanitin for 2 h followed with 10 nM E2 treatment to carry out the kinetic ChIP assay. A single chromatin was prepared for ChIP assay at each time point. (DOCX) [file pone.0166090.s004.docx]

**S4 Fig**


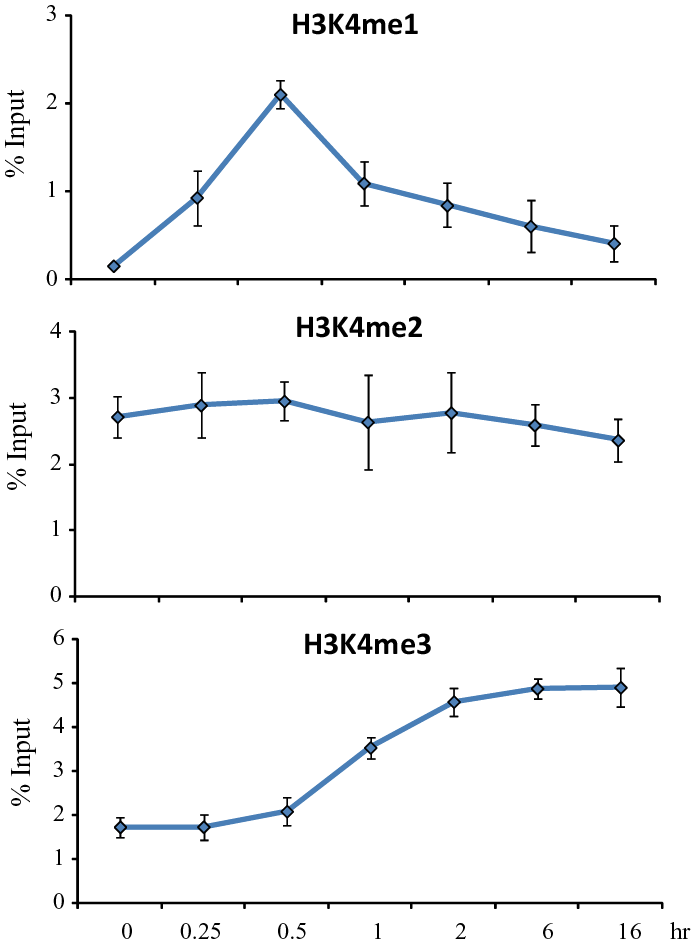


**S4 Fig.** **Kinetic ChIP experiments were performed using H3K4me1, H3K4me2 and H3K4me3 specific antibodies.** Cells were treated with 2.5 mM α-amanitin for 2 h followed with 10 nM E2 treatment to carry out the kinetic ChIP assay. A single chromatin was prepared for ChIP assay at each time point.
