# Supplementary material for: Regulation of IL-20 Expression by Estradiol through KMT2B-Mediated Epigenetic Modification
Source: PLoS One. 2016 Nov 2;11(11):e0166090. doi: 10.1371/journal.pone.0166090 (PMC5091760; doi:10.1371/journal.pone.0166090)
Supplement: S5 Fig — (DOCX) [file pone.0166090.s005.docx]

**S5 Fig**


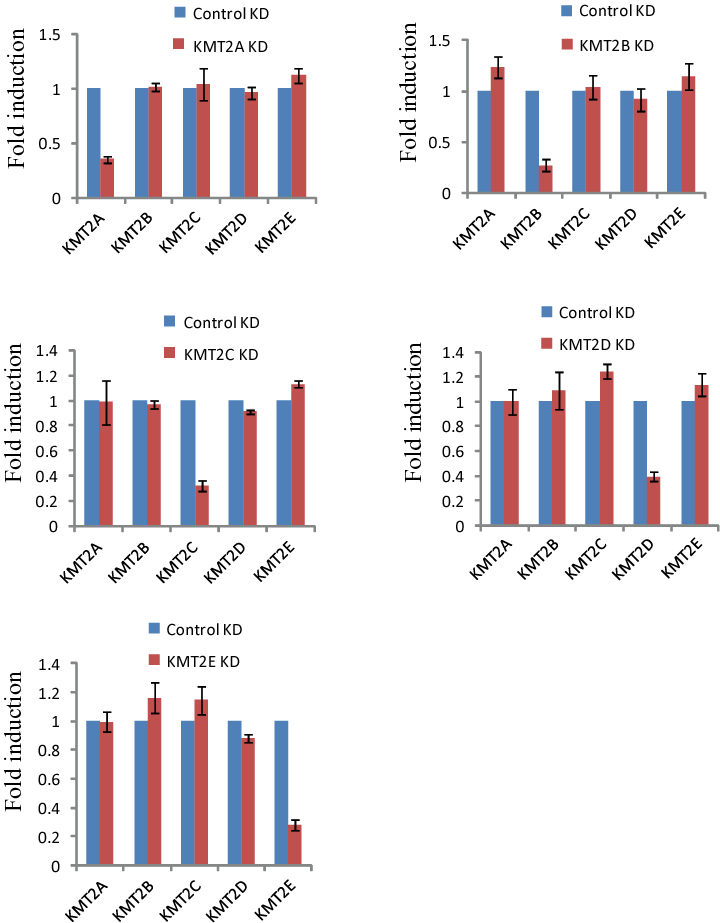


**S5 Fig.** Expression of KMTs was determined by RT-qPCR in KMT2A, KMT2B, KMT2C, KMT2D and KMT2E-depleted MCF-7 cells and normalized against 18s rRNA.
