# Supplementary figures and images for: Regulation of IL-20 Expression by Estradiol through KMT2B-Mediated Epigenetic Modification
Source: PLoS One. 2016 Nov 2;11(11):e0166090. doi: 10.1371/journal.pone.0166090 (PMC5091760; doi:10.1371/journal.pone.0166090)

**S6 Fig**


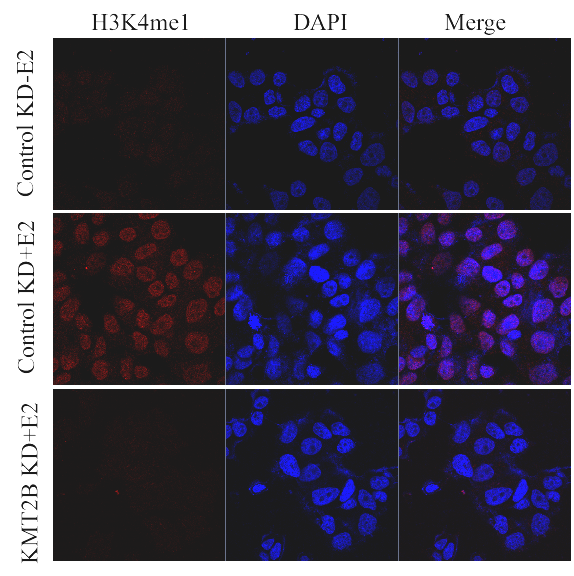


**S6 Fig.** H3K4me1 immunostaining (red) in Control KD or KMT2B KD cells in the presence of E2.

Supplement: S6 Fig — (DOCX) [file pone.0166090.s006.docx]
