# Supplementary material for: Regulation of IL-20 Expression by Estradiol through KMT2B-Mediated Epigenetic Modification
Source: PLoS One. 2016 Nov 2;11(11):e0166090. doi: 10.1371/journal.pone.0166090 (PMC5091760; doi:10.1371/journal.pone.0166090)
Supplement: S7 Fig — (A) A Venn diagram showing E2-stimulated genes down-regulated by KMT2B knockdown in MCF-7 cells. (B) Expression levels of PGR, BCL2, GREB1, SIAH2, and TFF1 in KMT2B-depleted MCF-7 cells with or without E2 for 4 h. Expression levels were normalized against 18S rRNA. (C) ChIP assay showing the effect of KMT2B depletion on the E2-dependent recruitment of ERα at BCL2, GREB1, and TFF1 chromatin. (D) ChIP assays showing the effect of ERα depletion on the E2-dependent recruitment of KMT2B at BCL2, GREB1, and TFF1 chromatin. ChIP assays showing the effect of ERα or KMT2B depletion on the enrichment of H3K4me1 mark (E) and the recruitment of RNA Pol II (F) at BCL2, GREB1, and TFF1 chromatin. (DOCX) [file pone.0166090.s007.docx]

**S7 Fig**


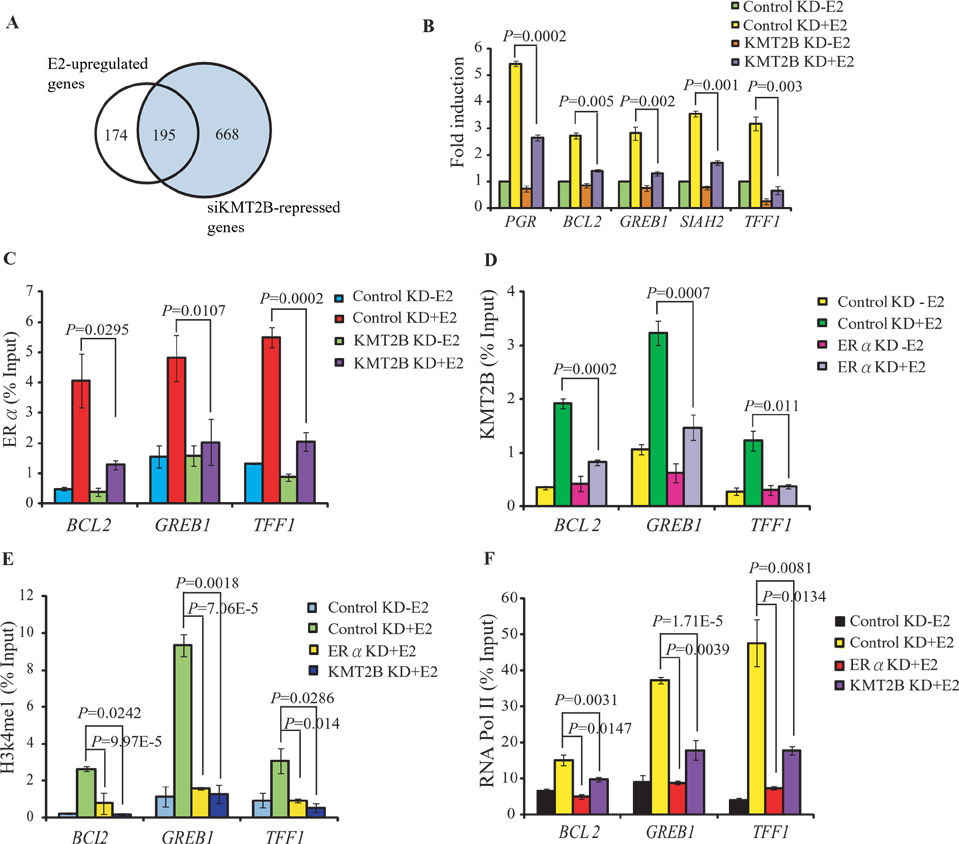


**S7 Fig. KMT2B regulates E2-dependent genes transcription in MCF-7 cells**. (A) A Venn diagram showing E2-stimulated genes down-regulated by KMT2B knockdown in MCF-7 cells. (B) Expression levels of *PGR*, *BCL2*, *GREB1*, *SIAH2*, and *TFF1* in KMT2B-depleted MCF-7 cells with or without E2 for 4 h. Expression levels were normalized against 18S rRNA. (C) ChIP assay showing the effect of KMT2B depletion on the E2-dependent recruitment of ERα at *BCL2*, *GREB1*, and *TFF1* chromatin. (D) ChIP assays showing the effect of ERα depletion on the E2-dependent recruitment of KMT2B at *BCL2*, *GREB1*, and *TFF1* chromatin. ChIP assays showing the effect of ERα or KMT2B depletion on the enrichment of H3K4me1 mark (E) and the recruitment of RNA Pol II (F) at *BCL2*, *GREB1*, and *TFF1* chromatin.
