# Supplementary material for: Regulation of IL-20 Expression by Estradiol through KMT2B-Mediated Epigenetic Modification
Source: PLoS One. 2016 Nov 2;11(11):e0166090. doi: 10.1371/journal.pone.0166090 (PMC5091760; doi:10.1371/journal.pone.0166090)
Supplement: S1 Table — (DOCX) [file pone.0166090.s008.docx]

**S1 Table**

**S1 Table.** Primers for qRT-PCR assay

| **Gene** | **Sequences** |
| --- | --- |
| *IL-20* | 5' CAAGACACAAAGCCTGCGAATC 3' |
|  | 5'  CTTCATTGCTTCCTCCCCACA 3' |
| *KMT2A* | 5' GGAGCGAGAGGAAAACAGCC 3' |
|  | 5'  CACCATAAGTCAAACATAACGCAC 3' |
| *KMT2B* | 5' CAGCCACCGCCGTCACCACA 3' |
|  | 5' GCTTCCACCGACTCATCTTCAG  3' |
| *KMT2C* | 5' AAGATGTCAACGGTCAGGAGTC 3' |
|  | 5'  ACTTCAATGTTTTCTGTCACTTCCA 3' |
| *KMT2D* | 5'  GCTGCCGTTGTCCCCATCT 3' |
|  | 5'  GAACACTGAGGAGGAAGGGGCT 3' |
| *KMT2E* | 5' CCAAGTACCCCAAAACAAAGAAG 3' |
|  | 5'  TGAAAGGAATGGTGAAGTAAATCTAA 3' |
| 18s rRNA | 5'GCCCGAAGCGTTTACTTTGA 3' |
|  | 5' TCCATTATTCCTAGCTGCGGTATC 3' |
| *BCL2* | 5' TGTGTGGAGAGCGTCAACCG 3' |
|  | 5' CTTTAGTGAACCTTTTGCATATTTGT 3' |
| *PGR* | 5' GGGCAATGGAAGGGCAGCAC 3' |
|  | 5' AACTCTGACTTTATTGAACTTTTT 3' |
| *GREB1* | 5' GGAACGTGGTGGATGTCAAC 3' |
|  | 5' CGTGGCAGCGGGAGAAGG3' |
| *SIAH2* | 5' CTGGCTATGGAGAAGGTGGC 3' |
|  | 5' TACTTCTCTTGTTTCTCCAGCACC 3' |
