# Supplementary material for: Regulation of IL-20 Expression by Estradiol through KMT2B-Mediated Epigenetic Modification
Source: PLoS One. 2016 Nov 2;11(11):e0166090. doi: 10.1371/journal.pone.0166090 (PMC5091760; doi:10.1371/journal.pone.0166090)
Supplement: S2 Table — (DOCX) [file pone.0166090.s009.docx]

**S2 Table**

|  |  |
| --- | --- |

**S2 Table.** Primers for chromatin immunoprecipitation assay

| **Gene** |  | **Sequences** |
| --- | --- | --- |
| *IL-20* | 1 | 5' ATTGGAAGTCAGAGAAGGGAGGG 3' |
|  |  | 5' TCTCCTGCTCTCTCCTCTAAACCC 3' |
|  | 2 | 5'  CCGTCATTGCCTTTCTTCTCTG 3' |
|  |  | 5' GGCATTTGTCTGAGATCAGATAGGTT 3' |
|  | 3 | 5'  GGGAGGCTTGGCAGTTTTTCTTAGT 3' |
|  |  | 5' AGAGAGAAGGCTGAAGGCAAGAC  3' |
|  | 4 | 5' CATCCTTGCTTGTTTTGTCTTCTTC  3' |
|  |  | 5' TGAGGGGAAAGGGGAGAGCAG 3' |
|  | 5 | 5' AGGATGGGTTGTGGAATAAGTTTTG 3' |
|  |  | 5' GGTTCCTACATCTTCTTCCTCAAA  3' |
|  | 6 | 5'  TCTTGCTCTGTTTCCCACGC 3' |
|  |  | 5'  GCTTCCTATCGCCCAGACTTT 3' |
| *BCL2* | 5' CACCTGTGGTCCACCTGAC 3' | |
|  | 5' CTGAAGAGCTCCTCCACCAC 3' | |
| *TFF1* | 5' GCTCCTTCCCTTCCCCCTGC 3' | |
|  | 5'  TCTCCTCCAACCTGACCTTAATCC  3' | |
| *GREB1* | 5' TGAGCAAAAGCCACAAAGTAGTTA  3' | |
|  | 5' TAAACCTGGATAACAAGAACACTG  3' | |
